# ZIF-L基硼酸亲和分子印迹材料的设计及其在环境水中利巴韦林检测中的应用

**DOI:** 10.3724/SP.J.1123.2025.04033

**Published:** 2026-02-08

**Authors:** Wanting QI, Yukui TONG

**Affiliations:** 哈尔滨师范大学化学化工学院，黑龙江 哈尔滨 150025; College of Chemistry and Chemical Engineering，Harbin Normal University，Harbin 150025，China

**Keywords:** 利巴韦林, 分子印迹聚合物, 吸附, 高效液相色谱法, ribavirin （RBV）, molecularly imprinted polymers （MIPs）, adsorption, high performance liquid chromatography （HPLC）

## Abstract

利巴韦林（ribavirin，RBV）作为一种广谱抗病毒药物，被广泛用于治疗多种病毒感染。然而，过量RBV进入水体将会对生态环境和人类健康造成严重危害。因此，开发一种针对环境水中RBV的简单、快速、高效的分析方法至关重要。本研究以二维ZIF材料（ZIF-L）为基质，RBV为模板分子，3-氨基苯硼酸（APBA）为功能单体，利用APBA自聚合能力在ZIF-L表面形成分子印迹，使用MeOH-HAc（1∶1，体积比）洗脱模板分子形成印迹空穴，从而构建了一种ZIF-L基硼亲和分子印迹聚合物（ZIF@B-MIPs）。利用pH响应型硼酸酯键形成动态识别位点，实现ZIF@B-MIPs与模板分子之间“形状记忆”与“化学键合”的协同识别。利用扫描电镜（SEM）和红外光谱仪（FT-IR）对ZIF@B-MIPs的尺寸形貌及表面基团进行了表征。系统考察了合成条件和吸附条件，优化条件下的实验结果显示，ZIF@B-MIPs对RBV的饱和吸附量达21.43 mg/g，其印迹因子（imprinting factor， IF）为5.32。吸附动力学符合拟二级模型（*R*^2^=0.995 3），15 min即达到吸附平衡，等温吸附符合Langmuir模型（*R*^2^=0.982 5）。此外，吸附实验及重复使用性实验表明该材料具有选择性识别能力和良好的重复利用性。在最佳萃取条件下，结合高效液相色谱（HPLC）技术，建立了一种测定环境水样中RBV的新方法。方法学验证结果表明，RBV在0.05~100 mg/L范围内具有良好的线性关系，检出限（LOD）和定量限（LOQ）分别为0.038 mg/L和0.081 mg/L。在实际环境水样检测中，加标回收率为83.8%~94.5%（RSD<2.1%）。该方法操作简便，稳定性好，为环境水体中抗病毒药物的快速筛查提供了有效策略。

## Abstract (en)

Ribavirin （RBV） is a broad-spectrum antiviral drug. It is widely used to treat various viral infections. However， its entry into water bodies can cause serious harm to both the ecological environment and human health. Thus， there is an urgent need for a simple and efficient method to detect RBV for detection purposes. In this study， a two-dimensional ZIF material （ZIF-L） served as the matrix， RBV as the template molecule， and 3-aminophenylboronic acid （APBA） as the functional monomer， using its self-polymerization ability to form a molecularly imprinted layer on the surface of ZIF-L. Imprinted cavities were created using a MeOH-HAc （1∶1， volume ratio） eluent to disrupt the interaction between RBV and APBA， yielding ZIF-L-based boronate affinity molecularly imprinted polymers （ZIF@B-MIPs）. Non-imprinted materials （ZIF@B-NIPs） were prepared identically without RBV. ZIF@B-MIPs features dynamic recognition sites formed through pH-responsive boronate ester bonds. This enabled synergistic recognition based on the template molecule’s “shape memory” and “chemical bonding”. Scanning electron microscopy （SEM） and Fourier-transform infrared spectroscopy （FT-IR） were employed to characterize the morphology and functional groups of the material. Notably， new FT-IR peaks emerged for ZIF@B-MIPs at 1 381 cm^-1^ （B-O vibration） versus ZIF-L， confirming APBA polymerization. This indicates that the polymerization of APBA onto the surface of ZIF-L was successful. The synthesis and adsorption conditions were optimized. The results showed that， with a ZIF-L dosage of 100 mg， the optimal amount of the self-polymerizing reagent APBA was 100 mg. A self-polymerization time of 5 h was sufficient to form an appropriately thick imprinted layer. At pH 8.5， the stability of the polymer network was maintained while the imprinting effect and mass transfer efficiency were maximized. The post-elution re-adsorption capacity retention rate reached 96.8%， balancing elution efficiency with structural integrity. A RBV mass concentration of 60 mg/L was selected for the experimental requirements. After optimization， the saturation adsorption capacity of ZIF@B-MIPs for RBV reached 21.43 mg/g. The imprinting factor （IF） was 5.32. The material’s performance was assessed through adsorption and reusability experiments. The results indicated that the material possesses good specificity and selective recognition ability. It also exhibits a rapid adsorption rate and good reusability. The adsorption experiment results showed that the adsorption kinetics followed the pseudo-second-order model （*R*^2^=0.995 3）. Adsorption equilibrium was achieved within 15 min. The adsorption process of RBV by the adsorbent involves chemisorption. The Langmuir isotherm adsorption model （*R*^2^=0.982 5） fitted the experimental data better. This indicates that the adsorption of RBV by ZIF@B-MIPs likely involves monolayer adsorption. In mixed solutions of RBV and three interfering substances （lamivudine，uridine，inosine） with mass concentration ratios of 1∶1 and 1∶10， the adsorption capacity of RBV by ZIF@B-MIPs remained high. The interfering substances had minimal impact on the adsorption performance. The reusability experiment results showed that the material retained 93.6% of its initial adsorption capacity after six adsorption-desorption cycles. The reproducibility of ZIF@B-MIPs was investigated using six batches of adsorbents. The adsorption capacity for RBV ranged from 19.41 to 20.73 mg/g. This indicates that the material also possesses good reproducibility. In the detection of actual environmental water samples， the method exhibited excellent applicability. The recoveries of RBV in environmental water samples at three spiked levels （30， 50， 60 mg/L） were 83.8%-94.5% （RSD<2.1%）. The detection system constructed in conjunction with high performance liquid chromatography （HPLC） demonstrated a good linear relationship in the range of 0.05 to 100 mg/L （*R*^2^=0.991 6）. The limit of detection was 0.038 mg/L （*S*/*N*=3）， and limit of quantification was 0.081 mg/L （*S*/*N*=10）. The ZIF@B-MIPs-HPLC technology was successfully applied to the efficient adsorption， enrichment， and detection of RBV in drinking water sources. It holds potential for applications in the fields of human health and environmental protection. This technology offers a new strategy for the rapid detection of trace antiviral drugs in environmental samples.

近年来，抗病毒药物的排放引起了人们的广泛关注^［1］^。利巴韦林（ribavirin，RBV）作为一种广谱抗病毒药物^［2］^，不仅适用于慢性丙肝、严重肾功能损害等由病毒引起的疾病的治疗，而且也被应用在治疗COVID-19和其他病毒感染上，RBV的全年使用量已经超过8 000吨^［3］^。如果含RBV的废液流入到环境中，会对环境造成危害，并且会通过食物或者水被人体摄入，间接影响人体健康，如致癌、造成胎儿畸形和溶血性贫血^［4］^。因此，迫切需要一种简单、快速，特异性的方法来分离分析RBV。分子印迹技术（molecular imprinting technology，MIT）具有高度的亲和力和特异性，可从复杂体系中精准地识别出目标分子，常用于选择性分离目标物质^［5］^。其中，沸石咪唑骨架（ZIF-L）是一种二维层状金属有机骨架（metal-organic framework，MOF）材料，可利用它良好的孔隙结构和较高的比表面积制备分子印迹聚合物（MIPs）^［6］^，还可以根据目标化合物的特点对ZIF-L进行改性或修饰来增强印迹材料对目标化合物的吸附性能。

硼亲和配体（如3-氨基苯硼酸，APBA）是吸附含顺式二醇类物质（如RBV）的常见及有效的功能单体^［7，8］^。在配体的硼酸基团中，硼原子具有空的*p*轨道，能够吸引电子，使得与硼原子相连的碳原子上的电子云密度降低，这种电子效应使得邻位的碳原子更容易失去氢原子；另外，在碱性条件下，APBA苯环上连接的氨基亲核性增强，很容易进攻另一个APBA分子上的硼酸基团邻位碳原子，从而产生聚合物网络^［9-11］^。因此，APBA可同时作为功能单体和自聚合单体，简化印迹过程，是一种合成识别RBV的MIPs材料的理想选择^［12］^。

基于此，本实验以ZIF-L为基质，预组装RBV模板分子，利用APBA兼具功能单体与自聚合单体的特性，直接在ZIF-L表面聚合形成硼酸亲和分子印迹聚合物（ZIF@B-MIPs）。该方法所得到的材料具有较快的吸附速率、较高的吸附容量和优异的选择竞争性。将得到的ZIF@B-MIPs应用于环境水样中RBV的检测，对RBV具有较宽的检测范围和较低的检出限。该方法前处理简单，普适性强，可为抗病毒药物环境风险监测提供检测依据。

## 1 实验部分

### 1.1 仪器与试剂

Milli-Q超纯水机（美国Millipore公司），PHSF-3F pH计（上海精密科学仪器有限公司），LC-20A高效液相色谱（HPLC，日本岛津），79-1磁力搅拌器（常州市国华仪器有限公司），KA-1000离心机（上海安亭科学仪器厂），ZK-82BB真空干燥箱（上海实验仪器有限公司），JSM 6700-F扫描电子显微镜（SEM），TENSOR Ⅱ傅里叶变换红外光谱（FT-IR，德国布鲁克公司），DOA-P504-BN泵（IDEX，USA）。

APBA（99%）、磷酸二氢钠（NaH_2_PO_4_，98%）、磷酸氢二钠（Na_2_HPO_4_，98%）、过硫酸铵（APS，98%）、氢氧化钠（NaOH，98%）、硝酸锌（Zn（NO_3_）_2_，98%）、磷酸（H_3_PO_4_，85%）、乙酸（HAc，99.5%）、乙醇（EtOH，98%）、甲醇（MeOH，98%）、乙腈（ACN，≥99.9%）购自上海化学试剂公司。过氧化氢（H_2_O_2_，30%）购自天津大茂化学试剂公司。RBV（98%）、拉米夫定（lamivudine，LMV，98%）、尿苷（uridine，URD，99%）、肌苷（inosine，IOS，98%）和2-甲基咪唑（2-methylimidazole，2-MI）均购自上海麦克林公司。实际环境水样取自哈尔滨松花江。

### 1.2 溶液配制

以Na_2_HPO_4_和NaH_2_PO_4_配制pH为8.5的磷酸盐缓冲液（PBS），在4 ℃下保存备用，所有溶液在HPLC检测之前均通过0.45 μm微孔滤膜过滤。

### 1.3 吸附剂的制备

#### 1.3.1 ZIF-L的制备

将0.5 g 2-MI溶解于10 mL超纯水中，室温下快速搅拌5 min，然后加入3 mL H_2_O_2_，搅拌2 min。将0.5 mL 0.1 g/mL Zn（NO_3_）_2_溶液与上述溶液混合，室温下静置4 h。将混合溶液离心，收集到的白色沉淀物经超纯水和EtOH洗涤3次，60 ℃真空干燥12 h，得到0.2 g ZIF-L^［13］^。

#### 1.3.2 ZIF@B-MIPs的制备

ZIF@B-MIPs的制备过程如下：室温下，将100 mg ZIF-L和10 mg RBV分散在10 mL PBS溶液（pH 8.5）中搅拌1 h，再加入100 mg APBA搅拌至溶解，随后，加入500 μL APS溶液（15 mg/mL）引发反应并连续搅拌5 h。将得到的产物离心分离，固体物质用10 mL MeOH-HAc（1∶1，体积比）洗脱去除模板分子，制备得到ZIF@B-MIPs。

非印迹分子聚合物（ZIF@B-NIPs）的制备除不添加RBV外，其他步骤同上。

### 1.4 色谱分析条件

色谱柱：Syncronis C18色谱柱（250 mm × 4.6 mm，5 μm，ThermoScientific，USA）；柱温：25 ℃。流动相（A）：0.05 mol/L NaH_2_PO_4_（pH 4.2）溶液和流动相（B）：ACN。梯度洗脱程序：0~5 min，5%B；5~8 min，5%B~30%B；8~12 min，30%B~5%B。流速：1.0 mL/min，进样体积10 μL。

### 1.5 材料的吸附性能考察

#### 1.5.1 动力学吸附性能

准确称取ZIF@B-MIPs/ZIF@B-NIPs 5 mg，加入到5 mL RBV溶液（60 mg/L，pH 8.5）中振荡吸附，取样时间分别为0.5、1、2、3、5、7、10、15、20、25、30 min，将吸附剂与溶液分离，上清液通过0.45 μm滤膜过滤，经HPLC测定，计算吸附量（*Q*）。吸附剂的吸附量和印迹因子（imprinting factor，IF）由下式计算：


*Q*=(*C*_0_-*C*)*V*/*m* （1）

IF=*Q*_ZIF@B-MIPs_/*Q*_ZIF@B-NIPs_ （2）

式中，*C*_0_和*C*（mg/L）分别为吸附前和吸附后溶液的浓度，*V*（L）为溶液的体积，*m*（g）为吸附剂的质量，*Q*_ZIF@B-MIPs_和*Q*_ZIF@B-NIPs_分别是ZIF@B-MIPs和ZIF@B-NIPs对RBV的吸附量（mg/g），IF是评价特定识别能力的指标，IF越高，表明分子印迹聚合物对模板分子的特异性吸附能力越强，印迹效果越好。

#### 1.5.2 等温吸附性能

准确称取5 mg ZIF@B-MIPs/ZIF@B-NIPs，分别加入到5 mL不同质量浓度（10、20、30、40、50、60、70、80 mg/L）的pH 8.5的RBV溶液中，室温下振荡15 min，将吸附剂与溶液分离，上清液通过0.45 μm滤膜过滤，经HPLC测定，计算吸附量。

#### 1.5.3 选择性与竞争性吸附性能

为了探究ZIF@B-MIPs/ZIF@B-NIPs对RBV的选择性识别能力，将RBV和3种干扰物（LMV、URD、ISO）配制成质量浓度比为1∶1的混合溶液（60 mg/L），取5 mg ZIF@B-MIPs/ZIF@B-NIPs分别加入5 mL该混合溶液中，在室温下振荡15 min后，将吸附剂与溶液分离，使用0.45 µm滤膜过滤，用HPLC测定RBV的含量。将干扰物质LMV、URD、ISO的浓度增大10倍，RBV浓度保持不变，重复上述操作，考察竞争性吸附性能。

## 2 结果与讨论

### 2.1 吸附剂的表征

#### 2.1.1 形貌分析

采用SEM表征了ZIF-L、ZIF@B-MIPs的形貌。如图1a所示，ZIF-L为花瓣形堆叠的薄片，总体呈现出花形结构^［13］^。图1b为ZIF@B-MIPs的SEM图像，由于MIPs的黏附效应，花瓣形状变得模糊，表面粗糙，可以看到由于APBA涂层的包裹，分子印迹材料的表面厚度增加。

**图1:**
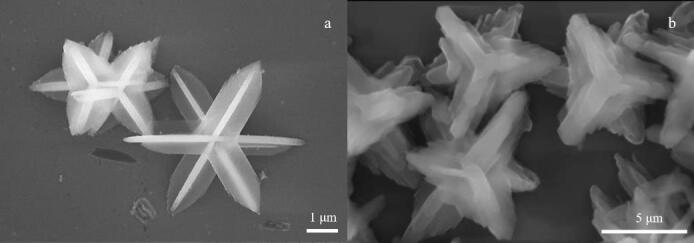
（a）ZIF-L及（b）ZIF@B-MIPs的扫描电镜图

为了探究印迹对吸附性能的影响，在同样实验条件下，分别计算两种材料的吸附量，ZIF@B-MIPs对模板分子RBV的吸附量（*Q*_ZIF@B-MIPs_=21.33 mg/g）显著高于ZIF-L（*Q*_ZIF-L_=3.76 mg/g），表明该分子印迹材料的合成增加了RBV的吸附位点，更有利于对RBV的吸附。

#### 2.1.2 红外光谱分析

FT-IR表征如图2所示，448 cm^-1^处的峰值归因于Zn-N的拉伸振动。691 cm^-1^、1 088 cm^-1^和1 230 cm^-1^处的峰分别代表面外和面内伸缩振动和弯曲振动。1 435 cm^-1^处的峰代表咪唑环上C=N键的伸缩振动。2 926 cm^-1^为咪唑环C-H键的拉伸振动^［14］^。3 410 cm^-1^归属于吸附剂中微量水分的O-H伸缩振动。与ZIF-L相比，ZIF@B-MIPs出现了新的特征吸收峰，1 381 cm^-1^处的特征吸收峰与B-O振动有关，表明APBA成功地在ZIF-L表面发生聚合，1 470 cm^-1^处的吸收峰归属于苯环上的C–H弯曲振动；ZIF@B-MIPs样品在1 590 cm⁻¹处的特征吸收峰与芳香环拉伸振动有关。

**图2:**
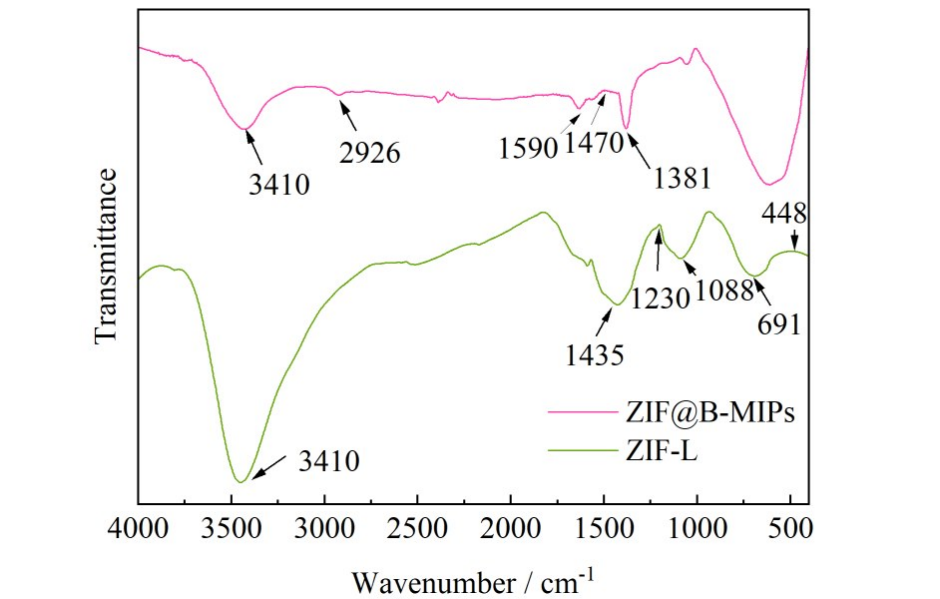
ZIF-L和ZIF@B-MIPs的红外光谱图

### 2.2 聚合条件的考察

#### 2.2.1 自聚合试剂比例的优化

为了探究最佳的合成条件，在ZIF-L用量（100 mg）不变的条件下，系统考察了APBA用量（60、70、80、90、100、110、120 mg）的影响。结果如图3a所示，随着APBA用量的增加，ZIF@B-MIPs对RBV的吸附量*Q*和IF呈现先增大后减小的趋势。当APBA用量小于100 mg时，随着APBA用量增加，所包覆的模板分子RBV数量增加，吸附剂的吸附能力增强，在100 mg时，*Q*和IF达到最大。但是，当APBA用量大于100 mg后，*Q*和IF逐渐下降，这可能是多余的APBA发生自聚合或印迹层过厚使包覆在内部的RBV不易完全洗脱所导致的。因此，确定APBA的最佳用量为100 mg。

**图3:**
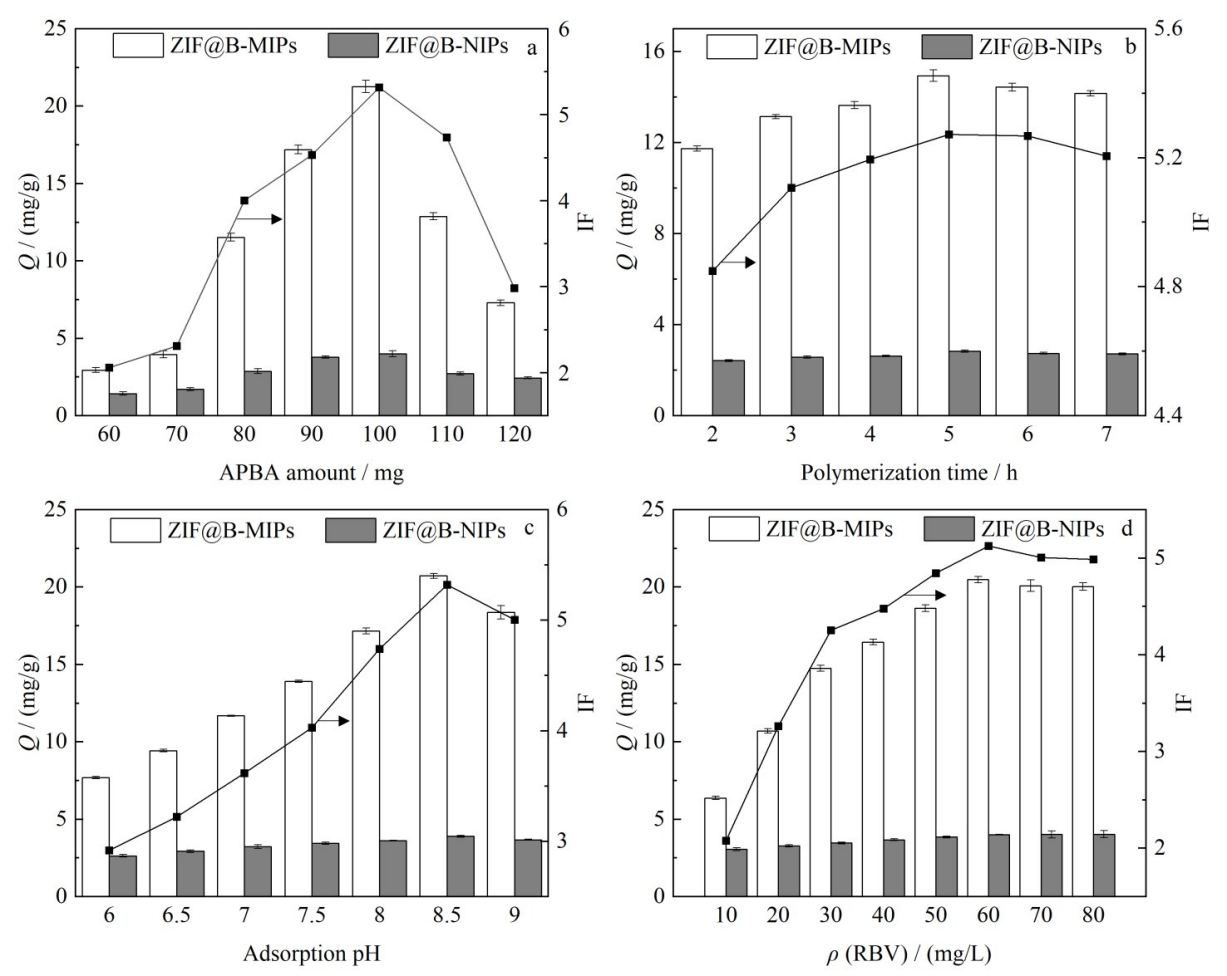
（a）APBA用量、（b）聚合时间、（c）吸附pH值以及（d）RBV的质量浓度对ZIF@B-MIPs和ZIF@B-NIPs吸附RBV的影响（*n*=3）

#### 2.2.2 自聚合时间的优化

自聚合时间对分子印迹材料的吸附性能也具有显著的调控作用。如图3b所示，随着聚合时间增加，*Q*和IF呈现先增大后略有减小的趋势。当自聚合时间达到5 h时，*Q*和IF达到最大，这归因于在此时间条件下形成的印迹层厚度适当。然而，当聚合反应时间大于5 h时，*Q*和IF减小，主要是因为聚合时间过长导致印迹层过厚，印迹位点包覆过深，在吸附过程中模板分子RBV不易到达空穴位点。因此，5 h为最佳聚合时间，既能保障印迹空穴的完整构筑，又可避免试剂浪费与性能衰减。

综上，吸附剂的最佳合成条件如下：APBA自聚合试剂的用量为100 mg，自聚合时间为5 h。

### 2.3 吸附条件的考察

#### 2.3.1 吸附pH的优化

为明确溶液pH对印迹材料吸附性能的影响，本研究通过调节RBV溶液pH（6.0~9.0）系统考察RBV的吸附行为。如图3c所示，随着pH由6.0升至8.5，IF值由2.92提升至5.32，*Q*呈现显著梯度增长（7.69~20.73 mg/g），这可能是因为弱碱性体系有利于硼酸配体和含顺式二醇的化合物形成稳定的环酯，而在酸性和中性环境中解离的硼酸阴离子四面体形式减少，硼酸酯化反应平衡移位。当pH大于8.5时，RBV的吸附量减少，这是由于含顺式二醇的分子和硼酸配体之间的静电排斥或者硼酸中的羟基解离造成的。因此，pH 8.5时吸附性能最优，故选择此pH进行后续实验。

#### 2.3.2 洗脱时间的优化

为了实现模板分子洗脱流程高效可控，本研究系统优化了洗脱时间对印迹材料性能的影响。采用MeOH-HAc（1∶1，体积比）作为洗脱剂时，洗脱效率表现出显著的时间依赖性：洗脱1 h模板分子洗脱率较低（69.6%），当洗脱时间延长至2 h，洗脱率有所上升（91.4%），继续洗脱至3 h时，洗脱率达98.8%。这种阶段式洗脱机制主要归因于溶剂分子的渗透动力学：初期（0~1 h）混合溶剂通过溶胀作用破坏聚合物网络中的*π*-*π*堆积；中期（1~2 h）HAc质子化作用解离功能单体与模板分子的氢键；后期（2~3 h）MeOH加速分子扩散以清除深层吸附位点。3次循环实验证实，3 h洗脱条件下材料再吸附容量保持率依旧能达到96.8%，在保证高效洗脱的同时维持了材料结构的稳定性。

#### 2.3.3 样品浓度的优化

本研究还考察了样品浓度对吸附量的影响，配制溶液质量浓度范围为10~80 mg/L，起初，每间隔10 mg/L测吸附量，*Q*值呈现上升趋势，但当浓度从60 mg/L增加到80 mg/L时，*Q*没有显著增加，IF也从峰值（5.13）开始下降（图3d），说明在60 mg/L时，印迹位点与RBV结合达到动态平衡。该过程表明，在吸附过程中，较高的目标物浓度有利于增加RBV与吸附位点的结合机会，但当质量浓度增加到一定程度时吸附位点饱和，吸附量变化不大，因此选择60 mg/L。

综上，该研究选择pH为8.5，洗脱时间为3 h，样品质量浓度为60 mg/L。

### 2.4 动力学和等温吸附性能分析

图4a为ZIF@B-MIPs和ZIF@B-NIPs的吸附动力学曲线。材料对RBV进行0.5~30 min的吸附，随着吸附时间增加，*Q*逐渐增加，这是因为ZIF@B-MIPs上的印迹位点逐渐与模板分子RBV结合。当吸附时间达到15 min时，ZIF@B-MIPs和ZIF@B-NIPs对RBV的*Q*达到最大且几乎不再变化，是因为吸附位点与RBV结合达到动态平衡。ZIF@B-MIPs的*Q*高于ZIF@B-NIPs，说明印迹层增加了印迹位点的数量。分别采用拟一级和拟二级动力学模型对实验数据进行拟合（图4c）。拟合结果表明，拟二级动力学模型（*R*^2^=0.995 3）能更好地描述吸附过程，表明ZIF@B-MIPs对RBV的吸附主要受化学作用控制。

**图4:**
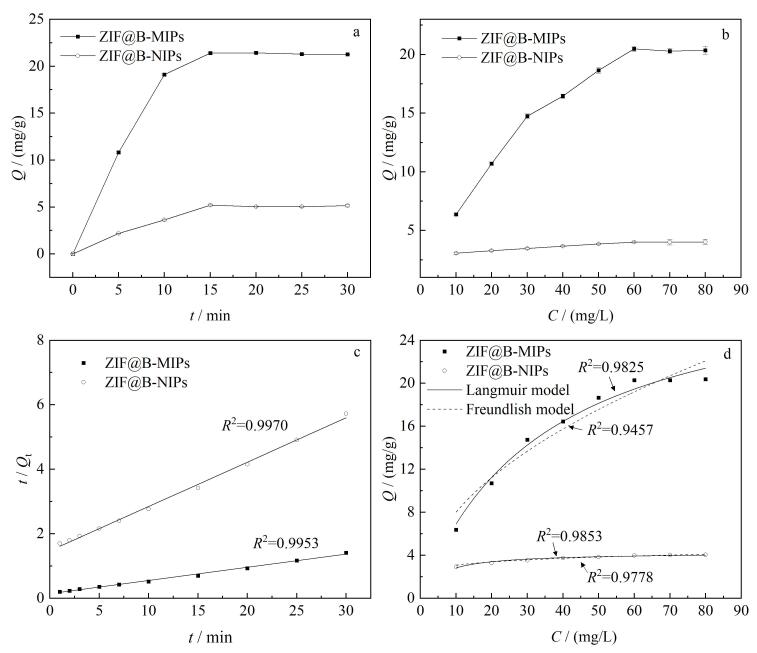
（a）吸附动力学图、（b）等温吸附图、（c）拟二级动力学拟合曲线以及（d）吸附热力学Langmuir模型和Freundlish模型拟合曲线（*n*=3）

图4b为ZIF@B-MIPs和ZIF@B-NIPs的等温吸附曲线。当RBV质量浓度达到60 mg/L时，材料吸附量达到最大，继续增大RBV浓度，*Q*几乎不变，此时ZIF@B-MIPs中印迹位点与RBV结合达到平衡。为了进一步探究等温吸附过程，选择Langmuir和Freundlich等温模型对等温数据拟合（图4d），结果表明，Langmuir等温吸附模型（*R*^2^=0.982 5）对实验数据的拟合度优于Freundlich模型，表明ZIF@B-MIPs对RBV的吸附更倾向于单层吸附机制。

### 2.5 选择性与竞争性

为了评价吸附剂对RBV的吸附性能，本研究选择3种结构类似物来评估ZIF@B-MIPs和ZIF@B-NIPs对RBV的特异性吸附能力。图5展示了ZIF@B-MIPs和ZIF@B-NIPs对RBV与干扰物混合溶液中选择性吸附前后的对比色谱图，并比较了各物质的*Q*和IF（图5a、5b）。ZIF@B-MIPs对RBV的*Q*高于ZIF@B-NIPs，这是由于分子印迹为RBV留下了特定的空腔位点，使得ZIF@B-MIPs能够特异性吸附RBV。同时，ZIF@B-MIPs对LMV吸附量比URD和IOS更小，这主要归因于识别机制的差异：LMV与RBV虽结构类似但缺乏顺式二醇基团，无法与硼酸形成共价键；URD和ISO虽含顺式二醇，但其分子尺寸和形状与RBV印迹空腔不匹配，导致识别能力显著低于RBV。

**图5:**
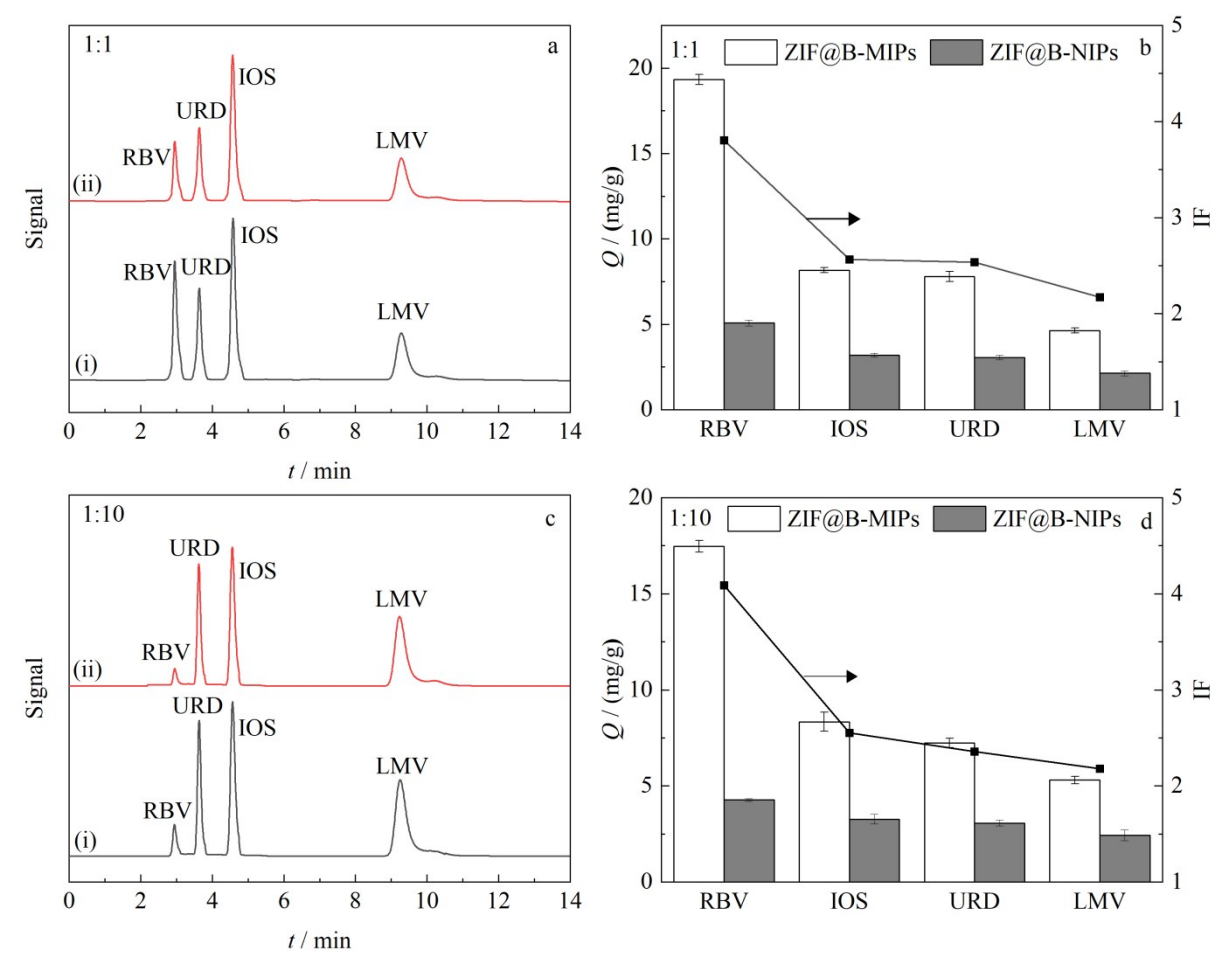
ZIF@B-MIPs对RBV和3种干扰物的（a）选择性和（c）竞争性吸附实验的色谱图，ZIF@B-MIPs/ZIF@B-NIPs对RBV及3种干扰物的（b）选择性和（d）竞争性吸附量及印迹因子对比（*n*=3）

RBV浓度不变，而其他竞争物的浓度扩大10倍后，从图5c和图5d中可以看出，3种物质干扰了ZIF@B-MIPs对目标物RBV的特异性识别，但在吸附量上总体趋势变化不大，ZIF@B-MIPs对RBV的IF仍保持在4.08。这表明，在高浓度干扰物的存在下，ZIF@B-MIPs仍能准确地吸附复杂混合溶液中的RBV，具有较高的选择性。

### 2.6 重现性与可重复使用性

优异的吸附剂应具有良好的重现性。因此，我们制备了6批次吸附剂，研究了材料的重现性。如图6所示，ZIF@B-MIPs对RBV的吸附量为19.41~20.73 mg/g，表明材料制备方法具有良好的重现性。使用同一批次吸附剂进行了6次连续的吸附-解吸循环，随着材料吸附-解吸次数增加，有一部分印迹位点因被模板分子RBV堵塞而难以洗脱，导致ZIF@B-MIPs对RBV吸附量减小，结果表明，6次循环后吸附量为初始吸附量的93.6%，说明材料具有良好的重复利用性。

**图6:**
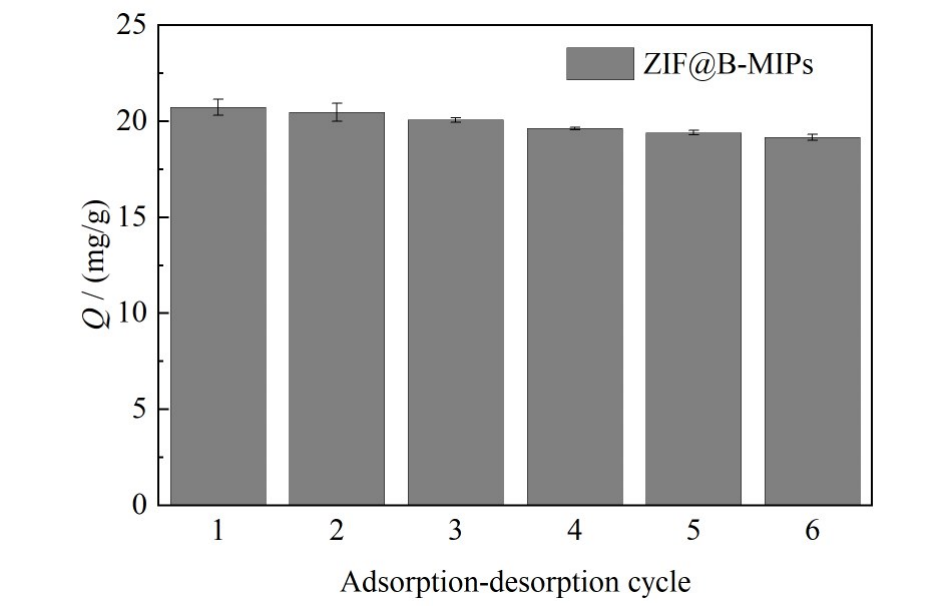
ZIF@B-MIPs和ZIF@B-NIPs的重复使用性（*n*=3）

### 2.7 线性范围、检出限和定量限

配制一系列质量浓度的RBV标准溶液，经ZIF@B-MIPs吸附及洗脱后，用0.45 µm滤膜过滤，在色谱条件下进行分析。扣除空白样品背景后，以峰面积为纵坐标（*y*）、质量浓度为横坐标（*x*，mg/L）进行线性拟合。实验结果表明，该方法对RBV检测的线性范围为0.05~100 mg/L，线性相关系数（*R*^2^）为0.991 6，分别以3倍信噪比和10倍信噪比确定检出限（LOD）为0.038 mg/L，定量限（LOQ）为0.081 mg/L。将该方法的LOD、LOQ等相关参数与其他文献报道对比，如表1所示。APBA单体与RBV模板分子通过硼酸酯键形成的三维印迹空穴展现出显著特异性^［15-17］^。

**表1:** 本方法与其他文献报道方法的比较

Adsorbents	Method	RSD/%	LOD/（mg/L）	LOQ	Ref.
MSPE^a^	HPLC-UV	-	-	2.68 μg/L	［^15^］
MIP-SPE^b^	HPLC-UV	0.265	0.82	2.72 mg/L	［^16^］
C@H@B-MIPs	HPLC-UV	0.8-2.5	0.023	0.076 mg/L	［^17^］
ZIF@B-MIPs	HPLC-UV	0.3-2.1	0.038	0.081 mg/L	this work

a. Zr-Fe_3_O_4_； b. molecularly imprinted polymers.

### 2.8 实际样品分析

针对RBV的痕量检测需求，本研究系统考察了ZIF@B-MIPs材料在复杂水基质中的实际应用性能。首先，通过HPLC直接分析确认本地采集的自来水（市政供水系统）和江水（松花江流域地表水）中RBV本底浓度均低于方法定量限。随后，进行加标回收试验以评估方法在实际基质中的准确性：向5 mL水样中分别添加30、50、60 mg/L RBV标准品构建模拟污染体系，每个浓度梯度设置3组平行样品（*n*=3）。加入5 mg ZIF@B-MIPs后，利用前述的吸附过程和洗脱条件进行吸附和洗脱，完成目标物富集。HPLC检测数据显示（表2），自来水和江水样品的加标回收率为83.8%~94.5%，所有结果均符合EPA 8000D对有机污染物检测的质量控制要求。如图7所示，材料可以将样品中的RBV吸附并利用洗脱剂洗脱，从而达到去除水体中RBV的目的。值得注意的是，相较于传统MIPs材料，ZIF@B-MIPs的介孔-微孔分级结构显著提升了传质效率，使其在保留高选择性（IF=5.32）的同时缩短检测时间。此特性表明该材料可有效克服天然水体中其他干扰物的竞争吸附效应，为现场快速检测提供了技术支撑。

**表2:** 不同样品中RBV在3个水平下的加标回收率（*n*=3）

Sample	Spiked/（mg/L）	Detected/（mg/L）	Recovery/%	RSD/%
Tap water	30	28.35	94.5	1.3
	50	46.65	93.3	0.6
	60	52.32	87.2	0.3
River water	30	25.14	83.8	2.1
	50	42.25	84.5	1.1
	60	51.42	85.7	0.4

**图7:**
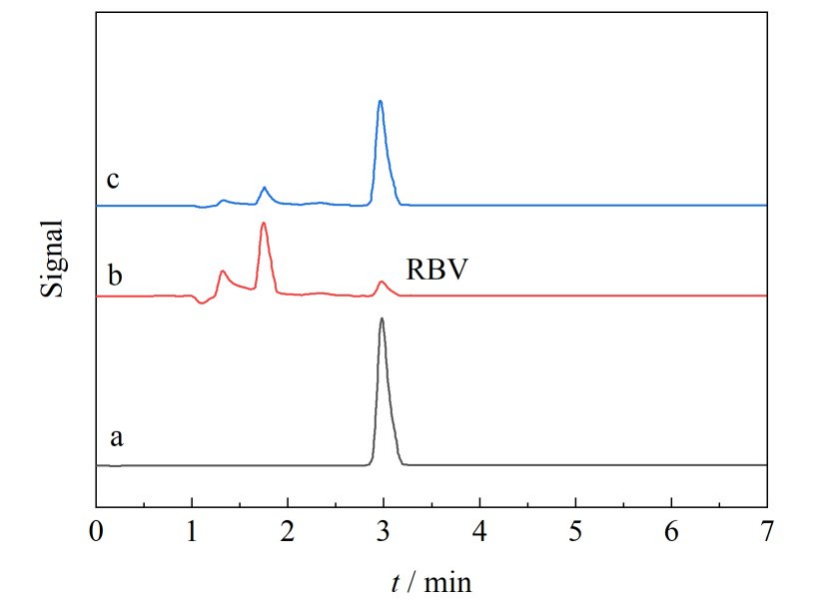
（a）RBV标准溶液、松花江水样加标（30 mg/L RBV）经ZIF@B-MIPs富集后（b）剩余溶液与（c）MeOH-HAc洗脱液的色谱图

## 3 结论

本研究成功构建了一种基于二维ZIF-L基质和APBA自聚合特性的ZIF@B-MIPs分子印迹材料，用于检测环境水中的RBV。实验优化结果表明，该吸附材料对RBV具有较高的吸附容量（*Q*=21.43 mg/g）、优异的印迹因子（IF=5.32）和较快的吸附平衡时间（15 min），吸附过程符合拟二级动力学和Langmuir等温模型。材料对RBV表现出显著的选择性，在高浓度干扰物存在下仍保持高识别能力（IF=4.08），并具备良好的重现性和重复使用性。通过与HPLC技术结合，建立了环境水样中RBV的分析方法。该方法线性范围宽，检出限低，对不同环境水基质的加标回收率较好（83.8%~94.5%）。本工作不仅为环境水体中痕量RBV的检测提供了一种高效可靠的方法，所开发的ZIF@B-MIPs材料在抗病毒药物环境监测领域展现出良好的应用潜力，也为开发基于分子印迹技术的现场快速检测装置奠定了材料基础。
